# Supplementary material for: Pimozide Increases a Delayed Rectifier K+ Conductance in Chicken Embryo Vestibular Hair Cells
Source: Biomedicines. 2023 Feb 8;11(2):488. doi: 10.3390/biomedicines11020488 (PMC9953418; doi:10.3390/biomedicines11020488)
Supplement: Supplementary file 1 [file biomedicines-11-00488-s001.zip › biomedicines-2141629-supplementary.pdf]

SUPPLEMENTARY MATERIAL

Table S1: Holding current at the membrane potential of -70 mV. Mean amplitude of the holding current at −70 mV from 15 type-II hair cells (E15-E21) before (CTRL) and after (PMZD) perfusion of pimozide [3 µM]. Values are shown as mean ± S.E.; the level of statistically significant difference is as follow: * $p \leq 0.05$; ** $p \leq 0.01$; *** $p \leq 0.001$; **** $p \leq 0.0001$.

**I holding**				
V_m	I (CTRL)	I (PMZD)		
−70 mV	−17.80 pA ± 6.95 pA	17.87 pA ± 11.80 pA	*	t = 3.364, df = 14, p-value = 0.0046<br>Student's paired t test

Table S2: Peak current values. Average peak current at each voltage steps obtained from 7 type-II hair cells (E15-E21) before (CTRL) and after (PMZD) perfusion of pimozide [3 µM]. Values are shown as mean ± S.E.; the level of statistically significant difference is as follow: * * $p \leq 0.05$; ** $p \leq 0.01$; *** $p \leq 0.001$; **** $p \leq 0.0001$.

**I peak**				
V_m	I (CTRL)	I (PMZD)		
−40 mV	386.47 pA ± 44.28 pA	527.58 pA ± 60.29 pA	*	W = 28, p-value = 0.0156<br>Wilcoxon signed-rank test
−30 mV	811.41 pA ± 99.57 pA	865.60 pA ± 94.99 pA	ns	W = 2, p-value = 0.9375<br>Wilcoxon signed-rank test
−20 mV	1272.65 pA ± 156.50 pA	1216.99 pA ± 130.89 pA	ns	t = 0.6308, df = 6, p-value = 0.5515<br>Student's paired t test
−10 mV	1722.23 pA ± 212.84 pA	1566.05 pA ± 160.13 pA	ns	t = 1.277, df = 6, p-value = 0.2489<br>Student's paired t test
0 mV	2196.99 pA ± 272.60 pA	1894.76 pA ± 189.50 pA	ns	t = 1.823, df = 6, p-value = 0.1181<br>Student's paired t test
+10 mV	2622 pA ± 307.72 pA	2219.83 pA ± 209.23 pA	ns	t = 1.927, df = 6, p-value = 0.1023<br>Student's paired t test
+20 mV	3067.92 pA ± 360.43 pA	2524.75 pA ± 209.49 pA	ns	t = 2.161, df = 6, p-value = 0.0740<br>Student's paired t test
+30 mV	3511.36 pA ± 407.29 pA	2834.11 pA ± 217.53 pA	ns	t = 2.142, df = 6, p-value = 0.0759<br>Student's paired t test
+40 mV	3955.97 pA ± 444.35 pA	3099.37 pA ± 220.97 pA	ns	t = 2.289, df = 6 , p-value = 0.0620<br>Student's paired t test

Table S3: Steady-state current values. Average steady-state current at each voltage steps obtained from 7 type-II hair cells (E15-E21) before (CTRL) and after (PMZD) perfusion of pimozide [3 μM]. Values are shown as mean ± S.E.; the level of statistically significant difference is as follow: * $p \leq 0.05$; ** $p \leq 0.01$; *** $p \leq 0.001$; **** $p \leq 0.0001$.

**I steady**				
V_m	I (CTRL)	I (PMZD)		
−40 mV	89.48 pA ± 13.29 pA	219.64 pA ± 29.53 pA	**	t = 4.716, df = 6, p-value = 0.0033 Student's paired t test
−30 mV	162.16 pA ± 20.05 pA	393.90 pA ± 23.68 pA	****	t = 9.381, df = 6, p-value < 0.0001 Student's paired t test
−20 mV	374.35 pA ± 32.02 pA	621.29 pA ± 27.10 pA	****	t = 9.892, df = 6, p-value < 0.0001 Student's paired t test
−10 mV	774.23 pA ± 69.96 pA	909.33 pA ± 56.03 pA	*	t = 3.326, df = 6, p-value = 0.0159 Student's paired t test
0 mV	1211.11 pA ± 113.78 pA	1197.01 pA ± 87.99 pA	ns	t = 0.2318, df = 6, p-value = 0.8244 Student's paired t test
+10 mV	1647.39 pA ± 141.31 pA	1484.03 pA ± 118.62 pA	*	t = 2.669, df = 6, p-value = 0.0371 Student's paired t test
+20 mV	2063.84 pA ± 170.09 pA	1746.45 pA ± 134.66 pA	**	t = 3.960, df = 6, p-value = 0.0075 Student's paired t test
+30 mV	2502.19 pA ± 203.25 pA	2006.61 pA ± 152.86 pA	**	t = 4.482, df = 6, p-value = 0.0042 Student's paired t test
+40 mV	2920.74 pA ± 232.42 pA	2251.01 pA ± 184.60 pA	**	t = 5.116, df = 6, p-value = 0.0022 Student's paired t test

Table S4: Normalized chord conductance values. Average normalized chord conductance values (mean ± S.E.) for $I_{K,v}$ at each voltage steps obtained from 7 type-II hair cells (E15-E21) before (CTRL) and after (PMZD) perfusion of pimozide [3 μM].

**G Steady**		
V_m	G Steady (CTRL)	G Steady (PMZD)
−70 mV	0.01 nS ± 0 nS	0.09 nS ± 0.03 nS
−60 mV	0.03 nS ± 0.01 nS	0.14 nS ± 0.04 nS
−50 mV	0.08 nS ± 0.02 nS	0.19 nS ± 0.04 nS
−40 mV	0.11 nS ± 0.02 nS	0.26 nS ± 0.05 nS
−30 mV	0.15 nS ± 0.03 nS	0.38 nS ± 0.04 nS
−20 mV	0.26 nS ± 0.03 nS	0.51 nS ± 0.04 nS
−10 mV	0.44 nS ± 0.03 nS	0.65 nS ± 0.03 nS
0 mV	0.60 nS ± 0.02 nS	0.76 nS ± 0.02 nS
+10 mV	0.73 nS ± 0.01 nS	0.85 nS ± 0.02 nS
+20 mV	0.83 nS ± 0.01 nS	0.91 nS ± 0.02 nS
+30 mV	0.93 nS ± 0 nS	0.96 nS ± 0.01 nS
+40 mV	1 nS ± 0 nS	1 nS ± 0 nS

Table S5: Peak current values. Average peak current at each voltage steps obtained from 8 type-II hair cells (E15-E19) before (CTRL) and after (PMZD) perfusion of pimozide [3 μM]. Values are shown as mean ± S.E.; the level of statistically significant difference is as follow: * $p \leq 0.05$; ** $p \leq 0.01$; *** $p \leq 0.001$; **** $p \leq 0.0001$.

**I peak**				
V_m	I (CTRL)	I (PMZD)		
−20 mV	539.52 pA ± 103.44 pA	682.59 pA ± 100.01 pA	***	t = 6.185, df = 7, p-value = 0.0005<br>Student's paired t test
−10 mV	988.71 pA ± 163.87 pA	1008.96 pA ± 119.32 pA	ns	W = 6, p-value = 0.7422<br>Wilcoxon signed-rank test
0 mV	1526.06 pA ± 227.45 pA	1399.66 pA ± 146.62 pA	ns	W = −16, p-value = 0.3125<br>Wilcoxon signed-rank test
+10 mV	2077.99 pA ± 287.71 pA	1748.17 pA ± 171.18 pA	*	t = 2.600, df = 7, p-value = 0.0354<br>Student's paired t test
+20 mV	2610.10 pA ± 340.39 pA	2063.99 pA ± 199.50 pA	**	t = 3.779, df = 7, p-value = 0.0069<br>Student's paired t test
+30 mV	3105.62 pA ± 382.41 pA	2373.31 pA ± 234.16 pA	**	t = 4.869, df = 7, p-value = 0.0018<br>Student's paired t test
+40 mV	3563.07 pA ± 402.63 pA	2679.53 pA ± 272.43 pA	***	t = 6.447, df = 7, p-value = 0.0004<br>Student's paired t test

Table S6: Steady-state current values. Average steady-state current at each voltage steps obtained from 8 type-II hair cells (E15-E19) before (CTRL) and after (PMZD) perfusion of pimozide [3 μM]. Values are shown as mean ± S.E.; the level of statistically significant difference is as follow: * $p \leq 0.05$; ** $p \leq 0.01$; *** $p \leq 0.001$; **** $p \leq 0.0001$.

**I steady**				
V_m	I (CTRL)	I (PMZD)		
−20 mV	492.26 pA ± 106.92 pA	609.39 pA ± 79.51 pA	**	t = 3.653, df = 7, p-value = 0.0082<br>Student's paired t test
−10 mV	921.68 pA ± 151.79 pA	871.26 pA ± 85.63 pA	ns	W = 2, p-value = 0.9453<br>Wilcoxon signed-rank test
0 mV	1385.18 pA ± 195 pA	1183.21 pA ± 109.12 pA	ns	W = −24, p-value = 0.1094<br>Wilcoxon signed-rank test
+10 mV	1834.60 pA ± 235.24 pA	1443.22 pA ± 123.45 pA	**	W = −36, p-value = 0.0078<br>Wilcoxon signed-rank test
+20 mV	2252.96 pA ± 274.91 pA	1689.52 pA ± 141.99 pA	**	t = 4, df = 7, p-value = 0.0052<br>Student's paired t test
+30 mV	2653.93 pA ± 303.84 pA	1917.82 pA ± 159.47 pA	**	W = −36, p-value = 0.0078<br>Wilcoxon signed-rank test
+40 mV	3019.70 pA ± 314.79 pA	2167.60 pA ± 190.81 pA	***	t = 6.601, df = 7, p-value = 0.0003<br>Student's paired t test

Table S7: Time-to-peak values. Average time-to-peak at each voltage steps. Data were obtained from 8 type-II hair cells (E15-E19) before (CTRL) and after (PMZD) perfusion of pimozide [3 μM]. Values are shown as mean ± S.E.; the level of statistically significant difference is as follow: * $p \leq 0.05$; ** $p \leq 0.01$; *** $p \leq 0.001$; **** $p \leq 0.0001$.

**Time to Peak**				
V_m	Time to Peak (CTRL)	Time to Peak (PMZD)		
−20 mV	210.29 ms ± 30.43 ms	106.61 ms ± 23.25 ms	**	t = 3.733, df = 7, p-value = 0.0073<br>Student's paired t test
−10 mV	149 ms ± 24.28 ms	86.63 ms ± 20.40 ms	**	W = −36, p-value = 0.0078<br>Wilcoxon signed-rank test
0 mV	105.61 ms ± 21.30 ms	55.78 ms ± 9.02 ms	*	t = 2.561, df = 7, p-value = 0.0375<br>Student's paired t test
+10 mV	72.18 ms ± 19.56 ms	46.22 ms ± 6.99 ms	ns	W = −24, p-value = 0.1094<br>Wilcoxon signed-rank test
+20 mV	65.55 ms ± 19.62 ms	36.11 ms ± 4.04 ms	ns	W = −28, p-value = 0.0547<br>Wilcoxon signed-rank test
+30 mV	56.34 ms ± 14.49 ms	35.70 ms ± 2.65 ms	ns	W = −26, p-value = 0.0781<br>Wilcoxon signed-rank test
+40 mV	52.07 ms ± 12.93 ms	32.84 ms ± 2.24 ms	ns	W = −22, p-value = 0.1484<br>Wilcoxon signed-rank test

Table S8: Inactivation time constant values. Average inactivation time constant (τ) at each voltage steps. Data were obtained from 8 type-II hair cells (E15-E19) before (CTRL) and after (PMZD) perfusion of pimozide [3 μM]. Values are shown as mean ± S.E.; the level of statistically significant difference is as follow: * $p \leq 0.05$; ** $p \leq 0.01$; *** $p \leq 0.001$; **** $p \leq 0.0001$.

**Inactivation time constant**				
V_m	τ (CTRL)	τ (PMZD)		
+10 mV	1127.88 ms ± 222.57 ms	759.75 ms ± 127.52 ms	*	t = 2.500, df = 7, p-value = 0.0410<br>Student's paired t test
+20 mV	1067.75 ms ± 318.08 ms	603.38 ms ± 84.42 ms	*	W = −32, p-value = 0.0234<br>Wilcoxon signed-rank test
+30 mV	890.13 ms ± 297.10 ms	573.75 ms ± 98.33 ms	ns	W = −16, p-value = 0.3125<br>Wilcoxon signed-rank test
+40 mV	799.75 ms ± 193.10 ms	554.88 ms ± 76.72 ms	ns	t = 1.748, df = 7, p-value = 0.1239<br>Student's paired t test

Table S9: Resting membrane potential at 0 pA. Average resting membrane potential measured in 7 type-II hair cells (E15-E20) before (CTRL) and after (PMZD) perfusion of pimozide [3 μM]. Values are shown as mean ± S.E.; the level of statistically significant difference is as follow: * $p \le 0.05$; ** $p \le 0.01$; *** $p \le 0.001$; **** $p \le 0.0001$.

**Resting membrane potential**			
V_m (CTRL)	V_m (PMZD)		
−42 mV ± 4.48 mV	−72 mV ± 2.65 mV	***	t = 7.113, df = 6, p-value = 0.0004 Student's paired t test

Table S10: Peak voltage values. Average peak voltage at each current steps obtained from 7 type-II hair cells (E15-E20) before (CTRL) and after (PMZD) perfusion of pimozide [3 μM]. Values are shown as mean ± S.E.; the level of statistically significant difference is as follow: * $p \le 0.05$; ** $p \le 0.01$; *** $p \le 0.001$; **** $p \le 0.0001$.

**V_m peak**				
I	V_m (CTRL)	V_m (PMZD)		
+10 pA	−40.65 ± 3.94 mV	−67.30 ± 2.44 mV	***	t = 6.715, df = 6, p-value = 0.0005 Student's paired t test
+60 pA	−32.97 mV ± 3.90 mV	−58.33 mV ± 3.45 mV	**	t = 5.545, df = 6, p-value = 0.0015 Student's paired t test
+110 pA	−28.24 mV ± 4.02 mV	−52.05 mV ± 3.67 mV	**	t = 4.753, df = 6, p-value = 0.0031 Student's paired t test
+160 pA	−24.76 mV ± 3.80 mV	−46.27 mV ± 4.24 mV	**	t = 4.420, df = 6, p-value = 0.0045 Student's paired t test
+210 pA	−20.66 mV ± 4.43 mV	−41.96 mV ± 4.12 mV	**	t = 4.298, df = 6, p-value = 0.0051 Student's paired t test
+260 pA	−17.19 mV ± 4.66 mV	−36.41 mV ± 4.28 mV	*	t = 3.679, df = 6, p-value = 0.0103 Student's paired t test

Table S11: Steady-state voltage values. Average steady-state voltage at each current steps obtained from 7 type-II hair cells (E15-E20) before (CTRL) and after (PMZD) perfusion of pimozide [3 μM]. Values are shown as mean ± S.E.; the level of statistically significant difference is as follow: * $p \le 0.05$; ** $p \le 0.01$; *** $p \le 0.001$; **** $p \le 0.0001$.

**V_m steady**				
I	V_m (CTRL)	V_m (PMZD)		
+10 pA	−36.77 mV ± 4.28 mV	−66.76 mV ± 2.66 mV	***	t = 7.218, df = 6, p-value = 0.0004 Student's paired t test
+60 pA	−27.84 mV ± 3.57 mV	−59.16 mV ± 3.17 mV	***	t = 6.794, df = 6, p-value = 0.0005 Student's paired t test
+110 pA	−23.46 mV ± 3.01 mV	−52.55 mV ± 3.73 mV	***	t = 6.521, df = 6, p-value = 0.0006 Student's paired t tes
+160 pA	−20.31 mV ± 2.73 mV	−46.20 mV ± 4.02 mV	**	t = 5.583, df = 6, p-value = 0.0014 Student's paired t test
+210 pA	−18.15 mV ± 3.00 mV	−41.84 mV ± 3.98 mV	**	t = 4.926, df = 6, p-value = 0.0026 Student's paired t test
+260 pA	−15.94 ± 2.87 mV	−36.23 mV ± 3.87 mV	**	t = 4.035, df = 6, p-value = 0.0068 Student's paired t test

Table S12: Peak current values. Average peak current at each voltage steps obtained from 7 type-II hair cells (E15-E21) before (CTRL) and after (PMZD) perfusion of pimozide [0.3 μM]. Values are shown as mean ± S.E.; the level of statistically significant difference is as follow: * $p \leq 0.05$; ** $p \leq 0.01$; *** $p \leq 0.001$; **** $p \leq 0.0001$.

**I peak**				
V_m	I (CTRL)	I (PMZD)		
−20 mV	299.65 pA ± 65.55 pA	825.92 pA ± 51.64 pA	***	t = 6.443, df = 6, p-value = 0.0007<br>Student's paired t test
−10 mV	734.55 pA ± 96.60 pA	1212.50 pA ± 69.96 pA	*	W = 28, p-value = 0.0156<br>Wilcoxon signed-rank test
0 mV	1222.67 pA ± 168.03 pA	1651.63 pA ± 98.45 pA	*	W = 28, p-value = 0.0156<br>Wilcoxon signed-rank test
+10 mV	1745.17 pA ± 238.33 pA	2077.69 pA ± 135.05 pA	ns	W = 18, p-value = 0.1563<br>Wilcoxon signed-rank test
+20 mV	2271.30 pA ± 310.70 pA	2484.33 pA ± 176.92 pA	ns	W = 10, p-value = 0.4688<br>Wilcoxon signed-rank test
+30 mV	2781.08 pA ± 381.84 pA	2900.77 pA ± 221.00 pA	ns	W = 2, p-value = 0.9375<br>Wilcoxon signed-rank test
+40 mV	3328.08 pA ± 474.60 pA	3314.46 pA ± 273.47 pA	ns	W = −10, p-value = 0.4688<br>Wilcoxon signed-rank test

Table S13: Steady-state current values. Average steady-state current at each voltage steps obtained from 7 type-II hair cells (E15-E21) before (CTRL) and after (PMZD) perfusion of pimozide [0.3 μM]. Values are shown as mean ± S.E.; the level of statistically significant difference is as follow: * $p \leq 0.05$; ** $p \leq 0.01$; *** $p \leq 0.001$; **** $p \leq 0.0001$.

**I steady**				
V_m	I (CTRL)	I (PMZD)		
−20 mV	267.66 pA ± 50.81 pA	770.58 pA ± 48.07 pA	***	t = 6.726, df = 6, p-value = 0.0005<br>Student's paired t test
−10 mV	704.87 pA ± 45.74 pA	1095.45 pA ± 60.67 pA	**	t = 5.908, df = 6, p-value = 0.0010<br>Student's paired t test
0 mV	1131.28 pA ± 73.19 pA	1417.06 pA ± 83.50 pA	**	t = 4.078, df = 6, p-value = 0.0065<br>Student's paired t test
+10 mV	1557.71 pA ± 111.35 pA	1725.25 pA ± 112.74 pA	ns	t = 2.303, df = 6, p-value = 0.0609<br>Student's paired t test
+20 mV	1978.70 pA ± 157.29 pA	2022.27 pA ± 146.77 pA	ns	t = 0.5297, df = 6, p-value = 0.6153<br>Student's paired t test
+30 mV	2381.15 pA ± 207.17 pA	2348.63 pA ± 188.48 pA	ns	t = 0.3739, df = 6, p-value = 0.7213<br>Student's paired t test
+40 mV	2815.10 pA ± 273.27 pA	2656.91 pA ± 227.98 pA	ns	t = 1.359, df = 6, p-value = 0.2229<br>Student's paired t test

Table S14: Time-to-peak values. Average time-to-peak at each voltage steps. Data were obtained from 7 type-II hair cells (E15-E21) before (CTRL) and after (PMZD) perfusion of pimozide [0.3 μM]. Values are shown as mean ± S.E.; the level of statistically significant difference is as follow: * $p \leq 0.05$; ** $p \leq 0.01$; *** $p \leq 0.001$; **** $p \leq 0.0001$.

**Time to Peak**				
V_m	Time to Peak (CTRL)	Time to Peak (PMZD)		
−20 mV	404.53 ms ± 101.86 ms	188.45 ms ± 61.70 ms	*	W = −24, p-value = 0.0469<br>Wilcoxon signed-rank test
−10 mV	152.73 ms ± 37.76 ms	115.97 ms ± 32.41 ms	ns	W = −10, p-value = 0.4688<br>Wilcoxon signed-rank test
0 mV	106.79 ms ± 30.28 ms	77.90 ms ± 25.98 ms	ns	W = −18, p-value = 0.1563<br>Wilcoxon signed-rank test
+10 mV	62.37 ms ± 14.43 ms	57.63 ms ± 15.19 ms	ns	W = −4, p-value = 0.8125<br>Wilcoxon signed-rank test
+20 mV	46.74 ms ± 7.15 ms	39.18 ms ± 6.46 ms	ns	W = −20, p-value = 0.1094<br>Wilcoxon signed-rank test
+30 mV	35.08 ms ± 4.25 ms	30.88 ms ± 2.14 ms	ns	t = 1.505, df = 6, p-value = 0.1830<br>Student's paired t test
+40 mV	29.92 ms ± 3.98 ms	31.35 ms ± 2.44 ms	ns	t = 0.4364, df = 6, p-value = 0.6778<br>Student's paired t test

Table S15: Inactivation time constant values. Average inactivation time constant (τ) at each voltage steps. Data were obtained from 7 type-II hair cells (E15-E21) before (CTRL) and after (PMZD) perfusion of pimozide [0.3 μM]. Values are shown as mean ± S.E.; the level of statistically significant difference is as follow: * $p \leq 0.05$; ** $p \leq 0.01$; *** $p \leq 0.001$; **** $p \leq 0.0001$.

**Inactivation time constant**				
V_m	τ (CTRL)	τ (PMZD)		
+10 mV	1159.61 ms ± 430.05 ms	907.84 ms ± 518.04 ms	ns	W = −6, p-value = 0.6875<br>Wilcoxon signed-rank test
+20 mV	912.24 ms ± 376.89 ms	543.96 ms ± 148.08 ms	ns	W = −10, p-value = 0.4688<br>Wilcoxon signed-rank test
+30 mV	697.30 ms ± 236.33 ms	509.67 ms ± 101.00 ms	ns	W = −12, p-value = 0.3750<br>Wilcoxon signed-rank test
+40 mV	463.27 ms ± 83.94 ms	449.75 ms ± 55.06 ms	ns	t = 0.2113, df = 6, p-value = 0.8397<br>Student's paired t test
